# Surface TLR2 and TLR4 Expression on Mature Rat Mast Cells Can Be Affected by Some Bacterial Components and Proinflammatory Cytokines

**DOI:** 10.1155/2011/427473

**Published:** 2011-05-02

**Authors:** Anna Pietrzak, Maciej Wierzbicki, Magdalena Wiktorska, Ewa Brzezińska-Błaszczyk

**Affiliations:** ^1^Department of Experimental Immunology, Medical University of Łódź, Pomorska 251, 92-213 Łódź, Poland; ^2^Department of Molecular and Medical Biophysics, Medical University of Łódź, Mazowiecka 6/8, 92-215 Łódź, Poland

## Abstract

The aim of our study was to determine whether some bacterial components as well as some proinflammatory cytokines can affect surface mast cell levels. By the use of flow cytometry technique, we documented that freshly isolated mature rat peritoneal mast cells do express surface TLR2 and TLR4 protein, but not CD14 molecules, and respond to stimulation with TLR2 and TLR4 ligands by cysteinyl leukotriene generation. The level of TLR2 protein is modulated by PGN and CCL5 treatment, but not by LPS, LAM, TNF, or IL-6. Surface mast cell TLR4 expression is affected by LPS, LAM, IL-6, and CCL5. Considering that TLR-mediated activation conditions not only engaged these cells in antibacterial defense and development of inflammation but also might influence allergic processes, our observations that surface TLR2 and TLR4 expression can be regulated both bacterial components and proinflammatory cytokines seem to be very intriguing and importance.

## 1. Introduction

Mast cells are found throughout connective tissues of the body and are particularly prevalent beneath the subepithelial surface of the skin, in the respiratory system, in the gastrointestinal and genitourinary tracts, and adjacent to blood vessels and nerves [1, 2]. Upon activation, these cells are capable of producing a wide range of mediators, including preformed, cytoplasmic granule-associated mediators, such as histamine, proteases, metalloproteinases and some cytokines, lipid mediators, including cysteinyl leukotrienes (cysLTs) and prostaglandins (PGs), and many *de novo* synthesized cytokines and chemokines. Mast cell-derived mediators can exert diverse proinflammatory, anti-inflammatory, and/or immunoregulatory effects [3]. That is why these cells take part not only in maintaining homeostasis [4] but also are important players in inflammatory processes [5, 6] and strongly influence both innate and adaptive immune responses [3, 7, 8]. Moreover, mast cells are involved in the development of many pathological processes [9, 10], inclusively allergic reactions [11, 12]. More and more data indicate that mast cells play crucial role in host defense, especially against bacteria, as well [13–15]. Considering enormous participation of mast cells in various pathophysiological processes, it seems to be of great importance to comprehend mast cell biology as well as to recognize the factors influencing mast cell activity.

It is without any doubt that toll-like receptors (TLRs), a class of pattern recognition molecules, play a crucial role in early host defense against invading microbes [16]. Several lines of evidence indicate that TLRs are implicated in inflammatory processes, as well [17, 18]. More and more data suggest the role of TLRs in the development of adaptive immunity [19]. TLRs are expressed on various types of immune cells. Different TLRs were demonstrated to be expressed by mast cells (reviewed in [20]), as well; however the regulation of mast cell TLR expression remain, as yet, poorly recognized. Taking into account that, on the one hand, mast cells express TLRs and, on the other hand, that these cells are significantly involved in the development of both innate and adaptive immunity and host defense mechanisms in this study, we determined whether some bacterial components, such as lipopolysaccharide (LPS) from *Pseudomonas aeruginosa*, lipoarabinomannan (LAM) from *Mycobacterium smegmatis*, and peptydoglican (PGN) from *Staphylococcus aureus* as well as some proinflammatory cytokines, that is, interleukin (IL)-6, tumor necrosis factor (TNF), and chemokine CCL5 (RANTES), could influence surface TLR2 and TLR4 levels on mast cells. 

## 2. Materials and Methods

### 2.1. Reagents

Dulbecco's Modified Eagle's Medium (DMEM), Hanks' balanced salt solution (HBSS), sodium bicarbonate, fetal calf serum (FCS), paraformaldehyde, gentamicin, and glutamine were obtained from GIBCO, Life Technologies (Gaithersburg, Md, USA). Percoll, toluidine blue, trypan blue, bovine serum albumine (BSA) N-2-hydroxyethylpiperazine-N′-ethanesulphonic acid (HEPES), NaCl, KCl, MgCl_2_, CaCl_2_, glucose, calcium ionophore A23187, sodium azide, and LPS from *P. aeruginosa* were obtained from Sigma-Aldrich Co. (St. Louis, Mo, USA). PGN from *S. aureus* and LAM from *M. smegmatis *were obtained from InvivoGen (San Diego, Calif, USA). Nuclear factor (NF)-*κ*B inhibitor—MG-132 was purchased from Biomol (Plymouth Meeting, Pa, USA). rr (rat recombinant) CCL-5, rrIL-6 and rrTNF were obtained from R&D Systems (Minneapolis, Minn, USA) and CysLT Immunoassay kit was purchased from Cayman Chemical (Ann Arbor, Mich, USA).

Rabbit anti-rat TLR2 IgG antibodies, rabbit IgG isotype control, phycoerythrin (PE)-conjugated donkey anti-rabbit IgG F(ab′)2 fragments, PE-conjugated mouse anti-rat TLR4 IgG antibodies, and PE-conjugates mouse IgG isotype control were obtained from Abcam Inc. (Cambridge, Mass, USA). Goat anti-rat CD14 IgG antibodies, goat IgG isotype control, fluorescein isothiocyanate (FITC)-conjugated donkey anti-goat IgG F(ab′)2 fragments, goat IgG antibodies blocking TLR2 and goat IgG antibodies blocking TLR4 were purchased from Santa Cruz Biotechnology (Santa Cruz, Calif, USA). 

### 2.2. Mast Cell Isolation

Mast cells were collected from peritoneal cavities of female albino Wistar rats weighing 200–250 g by lavage with 50 mL of 1% HBSS supplemented with 0.015% sodium bicarbonate. After abdominal massage (90 s), the cell suspension was removed from the peritoneal cavity, centrifuged (150 g, 5 min, 20°C), and washed twice in complete (c) DMEM containing DMEM supplemented with 10% FCS, 10 *μ*g/mL gentamicin, and 2 mM glutamine (150 g, 5 min, 20°C). To prepare purified mast cells, the peritoneal cells were resuspended in 72.5% isotonic Percoll solution and centrifuged (190 g, 15 min, 20°C). Next, isolated mast cells were washed twice in cDMEM by centrifugation. Purified mast cells were counted and resuspended in an appropriate volume of cDMEM or medium for rat mast cells containing 137 mM NaCl, 2.7 mM KCl, 1 mM MgCl_2_, 1 mM CaCl_2_, 10 mM HEPES buffer, 5.6 mM glucose, and 1 mg/mL BSA (pH of the medium was adjusted to 6.9). The mast cells were prepared with over 98% purity, as determined by metachromatic staining with toluidine blue. 

### 2.3. CysLT Release Assay

Purified mast cells were resuspended in appropriate volume of medium for rat mast cells to obtain mast cell concentration of 1.5 × 10^6^ cells/mL and incubated for 60 min at 37°C with LPS at final concentration of 10^2^ ng/mL, LAM or PGN at final concentrations of 10^2^ *μ*g/mL, calcium ionophore A23187 at final concentration of 5 *μ*g/mL (positive control) or buffer alone (spontaneous cysLT generation). The supernatants were collected by centrifugation (150 g, 5 min, 20°C) and analyzed by an ELISA commercial kit that detected LTC_4_ and its degradation products LTD_4_ and LTE_4_. The sensitivity of this assay was <13 pg/mL. 

To determine the specificity of bacterial antigen action on cysLT generation in some experiments, mast cells were preincubated with anti-TLR2 or anti-TLR4 antibodies (at final concentrations of 20 *μ*g/mL), with NF-*κ*B inhibitor MG-132 (at final concentration of 3 *μ*M) or medium alone for 15 min in a humidified atmosphere with 5% CO_2_ at 37°C. The cells were then washed twice, resuspended in medium and incubated with bacterial antigens, as described above. 

### 2.4. The Effect of Bacterial Components and Cytokines on TLR2 and TLR4 Expression

Purified mast cells resuspended in cDMEM were divided into 90 *μ*L aliquots (1.5 × 10 cells each). Subsequently, 10 *μ*L of LPS at final concentrations of 10^0^ ng/mL and 10^2^ ng/mL, PGN and LAM at final concentrations of 10^0^ *μ*g/mL and 10^2^ *μ*g/mL, CCL5, IL-6 or TNF (final concentrations of 1 pg/mL and 100 pg/mL), or cDMEM (control TLR2 and TLR4 expression on unstimulated mast cells) was added. Mast cells were incubated for 1, 3,  6,  12, and 24 h at 37°C in a humidified incubator with 5% CO_2_. After each period of incubation the cells were washed twice in 1% PBS (150 g, 4°C) and resuspended in 90 *μ*L of PBS. Additionally, after each period of incubation mast cell viability was examined by trypan blue exclusion assay. 

### 2.5. Flow Cytometry

For indirect TLR2 and CD14 staining, mast cells were fixed with 1% paraformaldehyde for 15 min, washed twice with 1% PBS (150 g, 4°C), resuspended in 90 *μ*L of 1% PBS and incubated with: (1) rabbit anti-rat TLR2 IgG antibodies or rabbit IgG isotype control and (2) goat anti-rat CD14 IgG antibodies or goat anti-rat IgG isotype control, respectively, at final concentrations of 1 *μ*g/10^6^ mast cells at 4°C for 45 min. After incubation the cells were washed in 1% PBS (150 g, 4°C) and incubated with PE-conjugated donkey anti-rabbit IgG F(ab′)2 fragments (TLR2 detection) or FITC-conjugated donkey anti-goat IgG F(ab′)2 fragments (CD14 detection) at final concentrations of 1 *μ*g/10^6^ mast cells at 4°C for 45 min in dark. For direct TLR4 staining, mast cells were suspended in ice cold 1% PBS supplemented with 1% FCS and 0.05% sodium azide and incubated with PE-conjugated mouse anti-rat TLR4 IgG antibodies or PE-conjugated mouse IgG isotype control at final concentrations of 1 *μ*g/10^6^ mast cells at 4°C for 60 min in dark. After incubation with antibodies/isotype control, the cells were washed twice with 1% PBS (150 g, 4°C) and finally resuspended in 300 *μ*L of 1% paraformaldehyde. Stained mast cell fluorescence was measured with a FACSCalibur flow cytometer with CellQuest software. The stimulated mast cell TLR2, TLR4, and CD14 expression was presented as percentage of TLR2, TLR4, and CD14 MFI (mean fluorescence intensity) measured in unstimulated mast cells (referred to as 100%).

### 2.6. Statistical Analysis

Statistical analysis included mean value, standard error of the mean (SEM), and Student's *t* test for “small groups”. Values of *P* < .05 were considered statistically significant. 

## 3. Results

### 3.1. Rat Peritoneal Mast Cells Express Functional TLR2 and TLR4 Molecules

**Figure 1:**
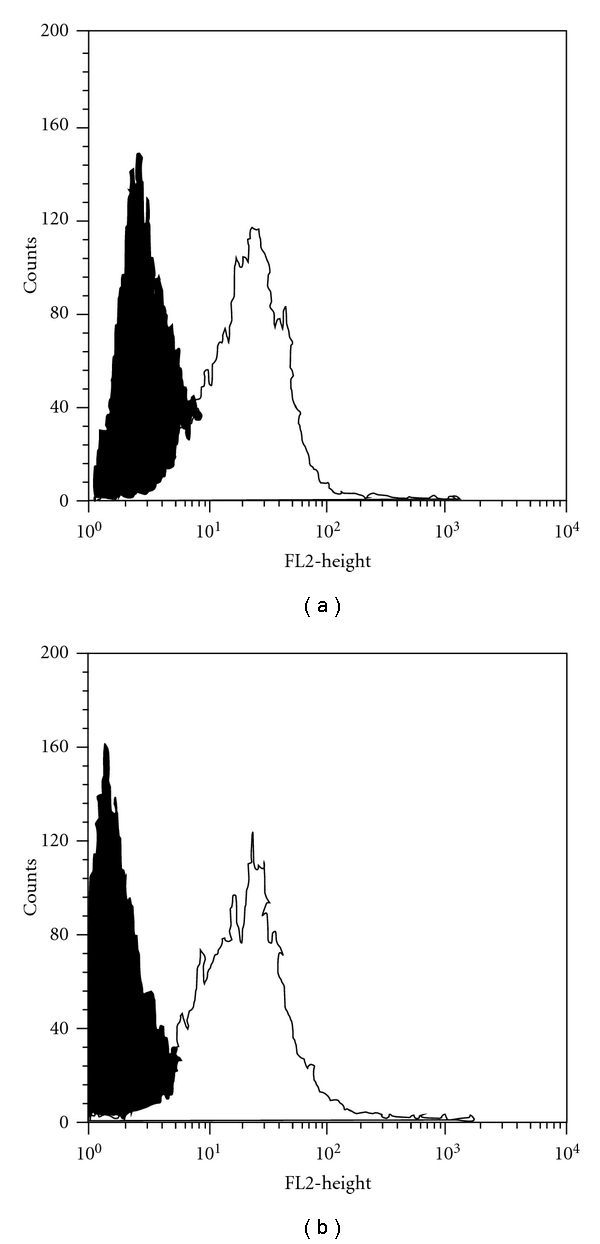
Representative flow cytometry histograms showing (a) TLR2 and (b) TLR4 expression on freshly isolated rat peritoneal mast cells. Filled histograms: isotype controls and open histograms: TLR expression.

We first examined the presence of TLR2, TLR4, and CD14 proteins on freshly isolated rat peritoneal mast cells by flow cytometry analysis. We stated that rat mast cells expressed both TLR2 and TLR4 molecules, and expression of surface TLR4 was higher than TLR2 level (Figure 1). We also found that these cells did not express CD14 protein (data not shown).

**Figure 2:**
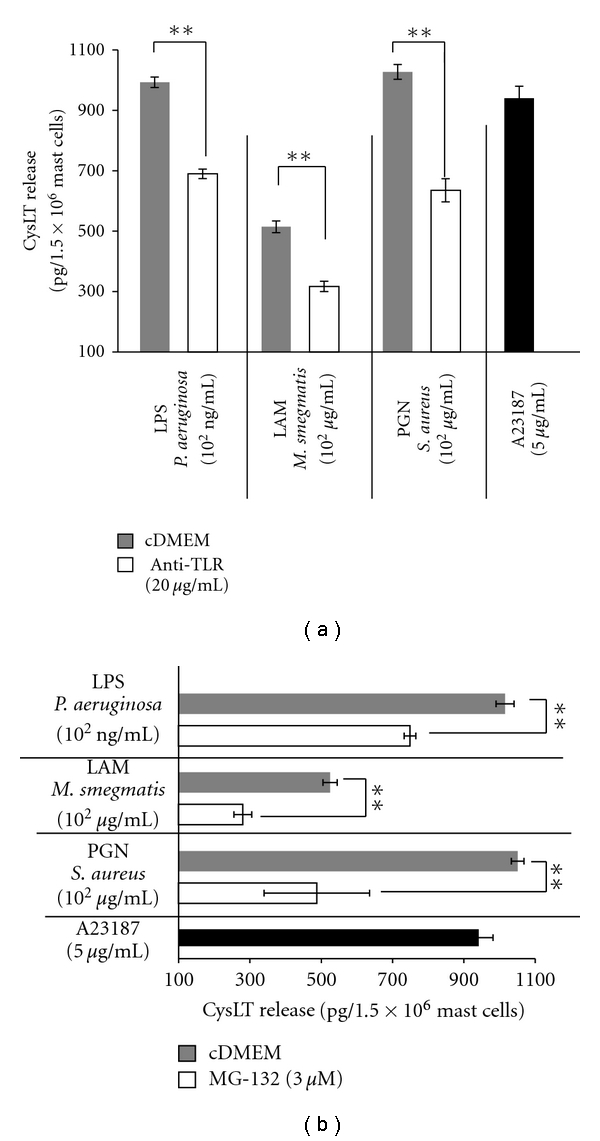
The effect of LPS, LAM and PGN on rat mast cell cysLT synthesis. (a) Mast cells were preincubated with cDMEM (control) or anti-TLR antibodies for 15 min and after washing incubated with LPS, LAM, PGN or buffer alone (spontaneous cysLT generation) for 60 min. Results are expressed as the mean ± SEM of three independent experiments and each experiment was done in duplicate (*n* = 6). (b) Mast cells were preincubated with cDMEM (control) or MG-132 for 15 min and after washing incubated with LPS, LAM, PGN or buffer alone (spontaneous cysLT generation) for 60 min. Results are expressed as the mean ± SEM of three independent experiments and each experiment was done in duplicate (*n* = 6). **P < .01.

We next investigated whether TLR2 ligands, that is, LAM and PGN, and TLR4 ligand, that is, LPS, would activate rat mast cells to generation and release cysLTs. We noticed that stimulation of mast cells with TLR4 agonist LPS for 60 min resulted in synthesis and release of significant amounts of cysLTs, as compared to ionophore A23187-induced cysLT generation. Mast cell activation with LAM and PGN, the TLR2 ligands, also caused generation and release of cysLTs; however the production of cysLTs in response to LAM was less robust. What is more, we observed that blocking TLR2 or TLR4 with related antibodies, prior to LAM, PGN or LPS challenge, strongly and statistically significant (*P* < .01) inhibited cysLT generation (Figure 2(a)). We also documented that preincubation of mast cells with MG-132, an NF-*κ*B inhibitor, caused a statistically significant (*P* < .01) decrease in LPS-, LAM-, and PGN-induced cysLT release (Figure 2(b)). 

### 3.2. Bacterial Components and Cytokines Can Modulate TLR2 Expression on Mast Cells

**Figure 3:**
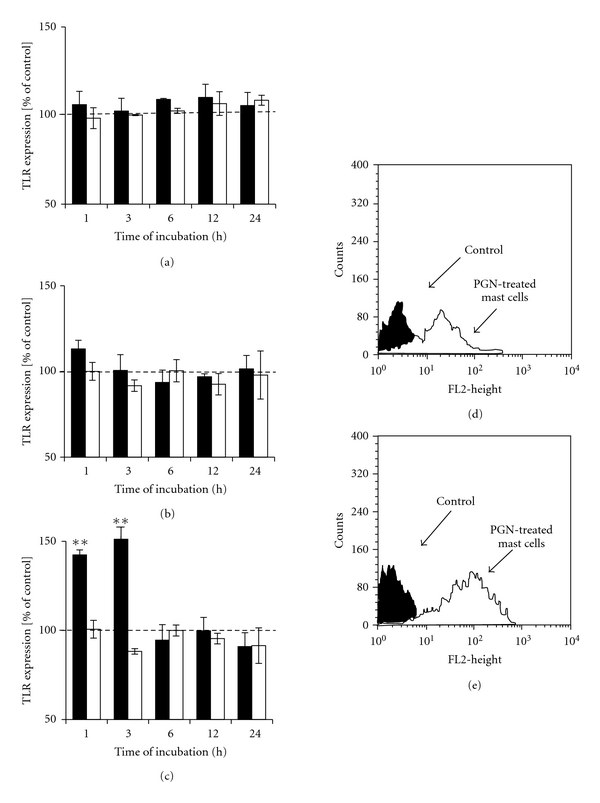
The effect of (a) LPS, (b) LAM, and (c) PGN on TLR2 expression on rat mast cells. Mast cells were incubated with bacterial components or cDMEM (control TLR2 expression on unstimulated cells referred to as 100%) for 1,  3,  6,  12, or 24 hours. LPS concentrations −1 ng/mL (dark bars) and 10^2^ ng/mL (bright bars) LAM and PGN concentrations −1 *μ*g/mL (dark bars) and 10^2^ *μ*g/mL (bright bars). Results are presented as a percentage of control TLR2 expression. Each point represents the mean ± SEM of 6 independent experiments. **P < .01. (d) Representative flow cytometry histogram showing TLR2 expression on rat mast cells treated with 1 *μ*g/mL PGN for 1 h. (e) Representative flow cytometry histogram showing TLR2 expression on rat mast cells treated with 1 *μ*g/mL PGN for 3 h. Filled histograms: untreated mast cells (control) and open histograms: PGN-treated mast cells.

**Figure 4:**
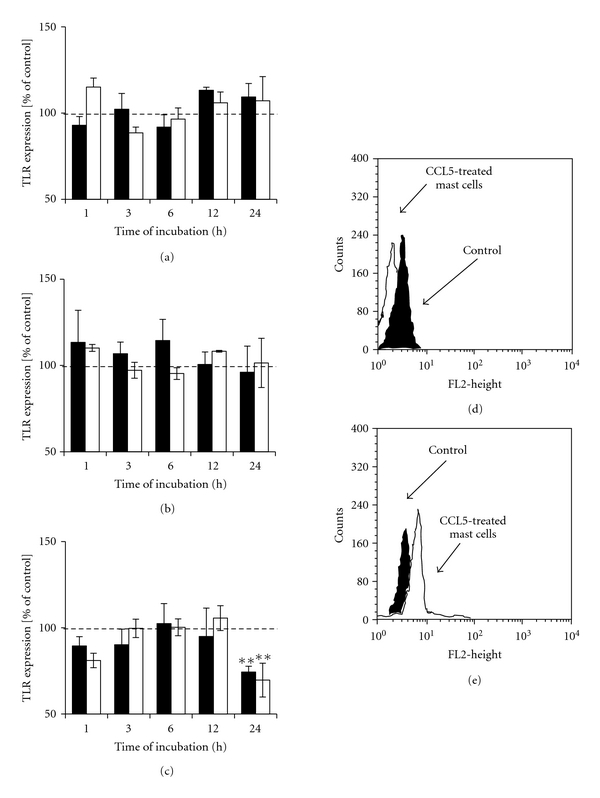
The effect of (a) TNF, (b) IL-6, and (c) CCL5 on TLR2 expression on rat mast cells. Mast cells were incubated with cytokines or cDMEM (control TLR2 expression on unstimulated cells referred to as 100%) for 1,  3,  6,  12, or 24 hours. Cytokine concentrations −1 pg/mL (dark bars) and 10^2^ pg/mL (bright bars). Results are presented as a percentage of control TLR2 expression. Each point represents the mean ± SEM of 6 independent experiments. **P < .01. (d) Representative flow cytometry histogram showing TLR2 expression on rat mast cells treated with 1 pg/mL CCL5 for 24 h. (e) Representative flow cytometry histogram showing TLR2 expression on rat mast cells treated with 10^2^ pg/mL CCL5 for 24 h. Filled histograms: untreated mast cells (control) and open histograms: CCL5-treated mast cells.

In order to examine the influence of LPS, LAM, and PGN as well as IL-6, TNF and CCL5 on surface TLR2 expression mast cells were incubated with each stimulant (or with cDMEM) for 1,  3,  6,  12, or 24 h and TLR2 level was estimated by flow cytometry analysis. We found that stimulation of mast cells with LPS used at concentrations of 10^0^ ng/mL  and 10^2^ ng/mL or LAM used at concentrations of 1 *μ*g/mL and 10^2^ *μ*g/mL for 1,  3,  6,  12, and 24 h did not affect the level of TLR2 protein (Figures 3(a) and 3(b)). Treatment of mast cells with 1 *μ*g/mL of PGN caused statistically significant (*P* < .01) increase in TLR2 expression level following 1 and 3 h incubation, as compared with the control unstimulated cells (Figures 3(c), 3(d), and 3(e)). We also established that mast cell TLR2 expression was not modulated by TNF or IL-6 treatment (Figures 4(a) and 4(b)); however, chemokine CCL5 at concentrations of 10^0^ pg/mL and 10^2^ pg/mL significantly (*P* < .01) downregulated the expression of TLR2 after 24 h incubation (Figures 4(c), 4(d), and 4(e)). 

### 3.3. Bacterial Components and Cytokines Can Modulate TLR4 Expression on Mast Cells

**Figure 5:**
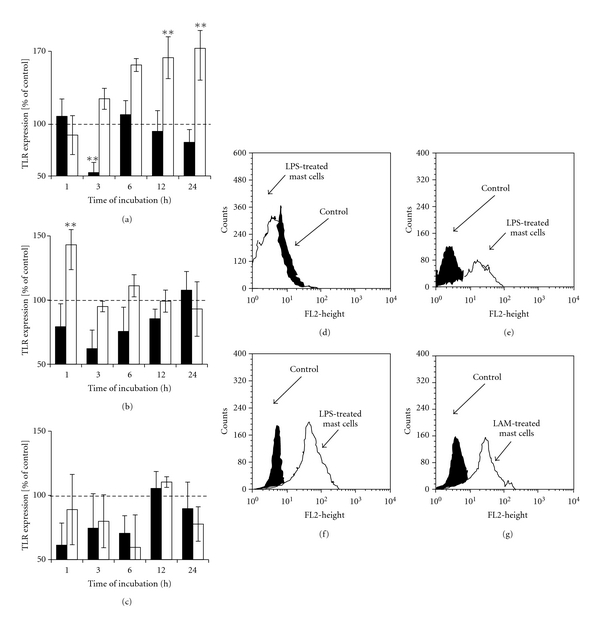
The effect of (a) LPS, (b) LAM, and (c) PGN on TLR4 expression on rat mast cells. Mast cells were incubated with bacterial components or cDMEM (control TLR4 expression on unstimulated cells referred to as 100%) for 1,  3,  6,  12, or 24 hours. LPS concentrations −1 ng/mL (dark bars) and 10^2^ ng/mL (bright bars) and LAM and PGN concentrations −1 *μ*g/mL (dark bars) and 10^2^ *μ*g/mL (bright bars). Results are presented as a percentage of control TLR4 expression. Each point represents the mean ± SEM of 6 independent experiments. **P < .01. (d) Representative flow cytometry histogram showing TLR4 expression on rat mast cells treated with 1 ng/mL LPS for 3 h. (e) Representative flow cytometry histogram showing TLR4 expression on rat mast cells treated with 10^2^ ng/mL LPS for 12 h. (f) Representative flow cytometry histogram showing TLR4 expression on rat mast cells treated with 10^2^ ng/mL LPS for 24 h. (g) Representative flow cytometry histogram showing TLR4 expression on rat mast cells treated with 10^2^ *μ*g/mL LAM for 1 h. Filled histograms: untreated mast cells (control) and open histograms: LPS- or LAM-treated mast cells.

We also studied the changes of TLR4 expression by mast cell treatment with LPS, LAM, and PGN. LPS at concentration of 1 ng/mL caused significant (*P* < .01) downregulation of TLR4 level following 3 h incubation, while the exposure of mast cells to 10^2^ ng/mL of LPS resulted in a statistically significant (*P* < .01) increased expression of TLR4 after prolonged 12 and 24 h incubation (Figures 5(a), 5(d), 5(e), and 5(f)). Mast cell treatment with 10^2^ *μ*g/mL LAM upregulated TLR4 expression following 1 h incubation (Figures 5(b) and 5(g)). PGN-treatment affected mast cell TLR4 expression, especially following 1,  3 and 6 h stimulation; however, the observed variations were not statistically significant (Figure 5(c)).

**Figure 6:**
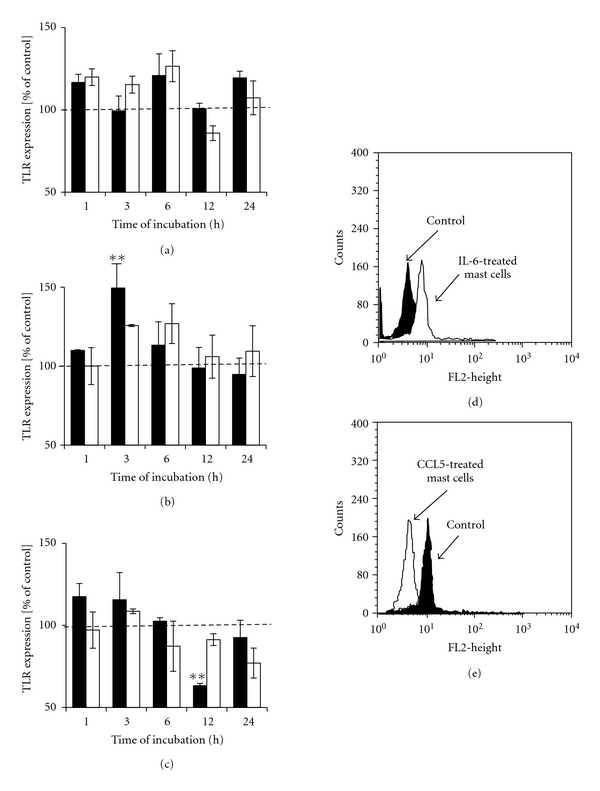
The effect of (a) TNF, (b) IL-6, and (c) CCL5 on TLR4 expression on rat mast cells. Mast cells were incubated with cytokines or cDMEM (control TLR4 expression on unstimulated cells referred to as 100%) for 1,  3,  6,  12, or 24 hours. Cytokine concentrations −1 pg/mL (dark bars) and 10^2^ pg/mL (bright bars). Results are presented as a percentage of control TLR2 expression. Each point represents the mean ± SEM of 6 independent experiments. **P < .01. (d) Representative flow cytometry histogram showing TLR4 expression on rat mast cells treated with 1 pg/mL IL-6 for 3 h. (e) Representative flow cytometry histogram showing TLR4 expression on rat mast cells treated with 1 pg/mL CCL5 for 14 h. Filled histograms: untreated mast cells (control) and open histograms: IL-6- or CCL5-treated mast cells.

The exposure of mast cells to TNF did not cause essential alteration in surface TLR4 level, at any time point examined, as shown in Figure 6(a). Treatment of mast cells with 1 pg/mL of IL-6 caused statistically significant (*P* < .01) increase in TLR4 expression level following 3 h incubation, as compared with the control unstimulated cells (Figures 6(b) and 6(d)), while stimulation of mast cells with 1 pg/mL CCL5 induced significant decrease in TLR4 level following prolonged 12 h incubation (Figures 6(c) and 6(e)). 

## 4. Discussion

It is well documented that mast cells express mRNA for TLR2 and TLR4 proteins. TLR2 and TLR4 mRNA expression was detected in both immature murine [21–25] and human [26–30] mast cells. The gene transcripts of TLR2 and TLR4 molecules were also found in mature murine [22] and human [26, 31] mast cells. Mast cell surface expression of TLR2 and TLR4 proteins has been sparsely estimated. TLR2 at the protein level was found on immature human [26–28, 30] mast cells as well as on mature mouse mast cells isolated from peritoneal cavity [24] and intestine [32], and mature nasal polyp human mast cells [33]. TLR4 protein was detected on immature human [27, 29, 30] and mouse [22] mast cells as well as on mature murine mast cells isolated from peritoneal cavity and skin [22]. To our knowledge, this study is the first report documenting TLR2 and TLR4 protein expression by mature freshly isolated rat peritoneal mast cells. It should be stressed that we stated that TLR4 level is higher than TLR2 expression. Moreover, we established that mast cells respond to stimulation with both PGN and LAM, that is, TLR2 ligands, as well as to LPS, that is, TLR4 ligand, by generation and release of cysLTs, according to previous reports [33–35], and this effect is partially, but significantly, decreased by anti-TLR2 or anti-TLR4 antibodies. What is more, by the use of flow cytometry technique, we also stated that rat peritoneal mast cells do not express CD14 molecules. Previously, McCurdy et al. [23] and Stassen et al. [36] demonstrated lack of CD14 protein expression on the surface of murine bone marrow-derived mast cells (BMMCs) and Varadaradjalou et al. [29] did not notice surface CD14 molecules on cord blood-derived mast cells (CBMCs). 

Considering the significance of TLR molecules in the development of both innate and adaptive immunity [37, 38] it seems to be of great importance to comprehend factors that influence TLR expression. It was indicated that some cytokines, including proinflammatory cytokines, can modulate the expression of TLR2 and/or TLR4 on macrophages [39], monocytes [40], endothelial cells [41], neutrophils [42], and epithelial cells [43]. Several reports described the effect of bacterial components on TLR expression, as well. It was shown that LPS upregulates the level of mRNA for TLR2, TLR4, and TLR9 in human monocytes and macrophages [44, 45] but decreases TLR4 mRNA expression in murine macrophages [46]. It was also documented that LPS stimulates increase in TLR2 and TLR4 expression on human airway epithelial cells [47] and elevation of mRNA for TLR4 in endothelial cells [41]. Hussain et al. [48] observed PGN-induced upregulation of TLR2 expression in murine pleural mesothelial cells, and Muzio et al. [45] documented LAM-induced increase in mRNA for TLR4 in human leukocytes. 

The impact of humoral factors and bacterial components on mast cell TLR expression is, as yet, not well recognized. Therefore, we decided to determine the influence of some important bacterial antigens, such as PGN—a main cell wall component of Gram-positive bacteria, LPS—an important component of Gram-negative bacteria, and LAM—a major virulence factor in the bacteria genus *Mycobacterium*, that is, TLR2 and TLR4 ligands, respectively, on TLR2 and TLR4 expression on mast cells. Taking into account that mast cells play a crucial role in inflammatory processes [5, 6], we also examined the effect of some proinflammatory cytokines, such as IL-6, TNF, and chemokine CCL5 on mast cell TLR2 and TLR4 levels. We observed that LPS mast cell treatment has no effect on the expression of TLR2 at any time point examined whereas it decreases TLR4 expression following 3 h incubation and increases TLR4 protein level following 12 and 24 h incubation. PGN treatment causes upregulation of TLR2 expression after 1 and 3 h incubation and LAM-treatment does not affect TLR2 expression but causes significant increase in TLR4 expression following 1 h incubation. So far, only Kubo et al. [27] examined the effect of bacterial components on TLR4 expression on mast cells. These authors stated that both LPS and LTA, but not PGN, stimulate augmentation of TLR4 mRNA in human mast cell lines LAD2 and HMC-1; however, only LPS induces an increase in TLR4 protein expression following 24 h incubation. It was also established that poly (I : C), a synthetic dsRNA construct, enhances TLR3 level in human and murine mast cells [31, 49]. We also documented that out of cytokines TNF does not modulate either TLR2 or TLR4 protein levels while IL-6 treatment of mast cells induces an increase in TLR4 expression following 3 h incubation. The exposure of mast cells to CCL5 resulted in decreased expression of both TLR2, following 24 h incubation, and TLR4 level, following 12 and 24 h incubation. Previously, Okumura et al. [50] found that TLR4 expression on human peripheral blood-derived mast cells is upregulated by interferon (IFN)-*γ*, and Yang et al. [51] stated that IL-12 induces significant increase in expression of TLR2 and TLR4 in P815 mast cell line. There was also documented that granulocyte-macrophage colony stimulating factor (GM-SCF) modulates expression of TLR3 and TLR7 in P815 cells [52] and cathelicidin LL-37 increases the level of TLR4 mRNA and TLR4 protein in mast cell line LAD2 [53]. 

The changes in TLR2 and TLR4 level on mast cells induced by bacterial components may be caused by a direct effect of ligands, that is, PGN or LPS, on its own receptors TLR2 and TLR4, respectively. However, observed modification of TLR expression could be mediated by an indirect mechanism involving cytokine production. It has been established that prolonged mast cell stimulation with bacterial cell wall components, such as LPS and PGN, resulted in *de novo* synthesis and release various cytokines [23, 25, 54, 55]. Thus, the increased expression of TLR4 following 12 and 24 h LPS stimulation could be resulted from cytokine activity. Unfortunately, we could not explain the exact mechanism of the IL-6 upregulation of TLR4 level and CCL5 downregulation of TLR2 and TLR4 expression.

Considering that TLR-mediated activation of mast cells conditions not only engaged these cells in antibacterial defense and development of inflammation but also might influence allergic processes [56, 57], our observations that mast cell surface TLR2 and TLR4 expression can be regulated by both bacterial cell wall components and proinflammatory cytokines seem to be very intriguing. However, further experiments are needed to clarify the mechanisms of modulation mast cell TLR2 and TLR4 expression by these factors.
